# Preventing Pressure Ulcers in the Operating Room: A National Survey of Operating Room Nurses' Knowledge and Attitudes

**DOI:** 10.1002/nop2.70808

**Published:** 2026-09-08

**Authors:** Karin Falk‐Brynhildsen, Carina Bååth, Dimitri Beeckman, Christine Leo Swenne, Charlotte Raepsaet, Camilla Wistrand

**Affiliations:** ^1^ Faculty of Medicine and Health, School of Health Sciences Örebro University Örebro Sweden; ^2^ Department of Health Sciences, Faculty of Health, Science and Technology Karlstad University Karlstad Sweden; ^3^ Department of Public Health and Primary Care, Ghent University Skin Integrity Research Group (SKINT), University Centre for Nursing and Midwifery Ghent Belgium; ^4^ Department of Public Health and Caring Sciences Uppsala University Uppsala Sweden; ^5^ University Health Care Research Center, Faculty of Medicine and Health Örebro University Örebro Sweden; ^6^ Department of Cardiothoracic and Vascular Surgery Örebro University Hospital Örebro Sweden

**Keywords:** APuP‐OR, attitudes, knowledge, operating room, operating room nurse, patient safety, pressure ulcers, PUKAT 2.0 OR

## Abstract

**Aim:**

To explore operating room nurses' self‐estimated knowledge and attitudes regarding the prevention of intraoperative pressure ulcers.

**Background:**

Pressure ulcers are a significant patient safety concern in surgical settings. Despite being largely preventable, intraoperative pressure ulcers continue to occur due to prolonged immobility, positioning and device‐related pressure. Nurses play a critical role in pressure ulcer prevention, yet knowledge gaps and unclear responsibilities may hinder effective practice.

**Design:**

A cross‐sectional study using a quantitative online survey conducted in accordance with STROBE guidelines.

**Methods:**

An online self‐reporting survey was distributed to 1904 eligible operating room nurses across Sweden. A total of 284 operating room nurses responded (response rate 15%). The survey included two validated instruments: the Pressure Ulcer Knowledge Assessment Tool—Operating Room (PUKAT 2.0 OR) and the Attitudes toward Pressure Ulcer Prevention—Operating Room (APuP‐OR). Descriptive and non‐parametric statistics were used to analyse the data.

**Results:**

Participants displayed solid knowledge of pressure ulcer aetiology, but their understanding of preventive strategies, particularly those related to intraoperative positioning, was markedly weaker. Attitudes towards prevention were consistently positive, and participants generally expressed confidence in their ability to prevent pressure ulcers. Nonetheless, a substantial proportion indicated a continued need for further education in preventive measures.

**Conclusion:**

Despite generally positive attitudes, operating room nurses' knowledge remains insufficient, especially regarding intraoperative positioning. Strengthening pressure ulcer prevention in surgical teams therefore requires structured educational initiatives and clearer role clarification of responsibilities within the surgical team. It is essential to enhance pressure ulcer prevention and improve patient safety.

**Implications for Nursing Practice:**

Operating room nurses' positive attitudes towards pressure ulcer prevention provide a strong foundation for practice improvement. Targeted evidence‐based education, competency assessments and enhanced interprofessional collaboration may strengthen preventive practices, reduce intraoperative pressure ulcers and improve patient safety during surgery.

**Patient or Public Contribution:**

There was no patient or public contribution.

## 1 Introduction

Pressure ulcers (PUs) represent a significant global challenge across healthcare settings, posing serious risks to patient safety and contributing to substantial societal costs. A comprehensive understanding of the multifactorial and interdisciplinary aetiology of PUs is essential for effective prevention, particularly in the context of intraoperatively acquired tissue damage. Insufficient knowledge in this area may increase the risk of PU development in surgical patients (Gefen et al. 2020).

The operating room (OR) is recognized as a high‐risk environment due to sustained exposure to mechanical forces that cause cellular and tissue deformation (European Pressure Ulcer Advisory Panel, National Pressure Injury Advisory Panel and Pan Pacific Pressure Injury Alliance 2019). All surgical patients are vulnerable to PU development, which is influenced by a complex interplay of systemic and environmental factors (Engels et al. 2016). In a recent study involving patients aged ≥ 65 years, the incidence of PUs was 18.8%. Variables such as gender, BMI, type of surgery, chronic disease and intraoperative blood transfusion were not identified as significant risk factors, whereas prolonged operative time (≥ 180 min) was strongly associated with increased PU risk (Uslu and Akbaba 2024). Identifying patient‐specific risk factors therefore remains a critical component of PU prevention strategies.

## 2 Background

According to the European Pressure Ulcer Advisory Panel (EPUAP), surgery‐related PUs occur in approximately 4%–45% of surgical procedures (European Pressure Ulcer Advisory Panel, National Pressure Injury Advisory Panel and Pan Pacific Pressure Injury Alliance 2019). Bulfone et al. (2012) reported an intraoperative PU incidence of 12.7%, with 46.1% of these ulcers persisting three days postoperatively. More recent studies have documented incidence rates ranging from 6.2% to 16.2% (İlkhan and Dag 2023; Tura et al. 2023). PUs typically manifests within 48 to 72 h post‐surgery due to sustained pressure exposure.

Medical devices used during surgery can further elevate PU risk, affecting both skin and mucosal surfaces. Device‐related PUs are associated with equipment such as spectral index monitors, blood pressure cuffs, adhesive tapes and endotracheal tubes (Choi et al. 2021; Pittman et al. 2015). Additionally, the type of mattress used during surgery plays a crucial role in PU development (Pittman et al. 2021; Yoshimura et al. 2016). Preventive interventions, including specialized mattresses, positioning pillows, and protective padding, aim to mitigate pressure, shear forces and prolonged immobility. Key intraoperative factors such as anaesthesia duration, blood loss, hemodynamic stability and oxygenation should be closely monitored (Weng and Chang 2023).

Recent data indicate that the prevalence of PUs related to medical devices is approximately 11.8%, while positioning‐related PUs may reach 23.5% (Özdemir et al. 2023). Thus, comprehensive knowledge of PU prevention is essential in the OR setting.

Gefen et al. (2020) emphasized the limitations of current risk assessment tools, highlighting the importance of clinical judgement in identifying PU risk. A systematic review by Lovegrove et al. (2023) found that clinical judgement alone lacks a standardized definition and predictive accuracy. Consequently, structured risk assessment tools should be considered the gold standard for PU evaluation.

EPUAP guidelines recommend pressure redistribution and micro‐repositioning during surgery, when feasible, based on the patient's condition (European Pressure Ulcer Advisory Panel, National Pressure Injury Advisory Panel and Pan Pacific Pressure Injury Alliance 2019). Operating room nurses (OR nurses) play a pivotal role in ensuring safe patient positioning and minimizing PU risk (Gefen et al. 2020). Defining formal responsibilities and processes for intraoperative positioning is essential (Brooker et al. 2020).

Studies by Sving et al. (2020, 2023) further support the limited predictive value of risk assessments, reinforcing the importance of clinical judgement. OR nurses should proactively review surgical schedules to identify procedures exceeding 2–3 h, as these patients are at elevated risk. Additionally, the American Society of Anaesthesiologists physical status classification can aid in identifying comorbidities that may influence PU risk (Gefen et al. 2020).

Knowledge gaps in PU assessment, prevention and treatment among OR nurses remain a barrier to effective PU management (Khong et al. 2020). Kaya and Ursavaş (2023) found that while OR nurses generally hold positive attitudes towards PU prevention, their knowledge is insufficient. Addressing these gaps is a critical first step in improving intraoperative care. Perioperative leaders should ensure that OR nurses possess the necessary knowledge and competencies to support safe patient positioning and continuity of care.

## 3 Purpose

The aim of this study was to describe Swedish OR nurses' self‐estimated knowledge and attitudes regarding the prevention of intraoperative PUs.

## 4 Methods

### 4.1 Design

This study employed a cross‐sectional design using a quantitative online survey. The manuscript was prepared in accordance with the STROBE (Strengthening the Reporting of Observational Studies in Epidemiology) guidelines for cross‐sectional studies and adhered to established scientific and ethical standards for publication.

### 4.2 Sample

OR nurses employed in one of Sweden's 21 healthcare regions were invited to participate. The inclusion criterion was being an OR nurse currently working in clinical practice or holding a managerial role. An online self‐administered survey was distributed via email to 2247 OR nurses. Of these, 343 email addresses were non‐functional, resulting in a final eligible sample of 1904 individuals. A reminder was sent one week after the initial distribution, followed by a second reminder prior to survey closure. In total, 284 OR nurses completed the survey, yielding a response rate of 15%.

### 4.3 Data Collection

Data collection was conducted between August and September 2020 as part of a larger research initiative focused on the development and validation of two instruments: the Pressure Ulcer Knowledge Assessment Tool—Operating Room (PUKAT 2.0 OR) and the Attitudes toward Pressure Ulcer Prevention—Operating Room (APuP‐OR).

The PUKAT 2.0 OR instrument comprises 14 multiple‐choice items, each with 5 response options including ‘I don't know the answer’. The items are categorized into 3 domains: *aetiology* (5 items; Cronbach's *α* = 0.56), *classification and observation* (2 items; *α* = 0.22) and *pressure ulcer prevention* (7 items; *α* = 0.74) (Falk‐Brynhildsen et al. 2023).

The APuP‐OR instrument includes 13 items rated on a 4‐point Likert scale and addresses 5 dimensions: *competence* (*α* = 0.67), *priority* (*α* = 0.50), *impact* (*α* = 0.44), *effectiveness* (*α* = 0.25) and *responsibility* (*α* = 0.50) (Falk‐Brynhildsen et al. 2022).

Additionally, the survey included one open‐ended question regarding participants' self‐perceived expertise in PU prevention, as well as demographic items covering age, gender, educational background and professional experience in healthcare.

### 4.4 Data Analysis

Descriptive statistics (frequency, percentage, mean and standard deviation) were used to summarize the sample characteristics. The Kruskal‐Wallis test with Bonferroni corrections was used to analyze the instrument responses. Questionnaires were reviewed for missing or duplicate entries.

Responses to the PUKAT 2.0 OR were recoded into a dichotomous variable (correct/incorrect), with ‘I don't know the answer’ categorized as incorrect. The APuP‐OR responses were scored on a 4‐point Likert scale (range: 13–52) and subsequently recoded into a dichotomous variable (agree/disagree) for analysis. Statistical analyses were performed using version 25 of IBM SPSS Statistics.

## 5 Results

### 5.1 Participant Demographics

A total of 284 OR nurses completed the online self‐reporting survey. The mean age of participants was 42.8 years (SD = 9.4). The majority were women (*n* = 264, 93%), with men comprising 7% (*n* = 20) of the sample. Regarding educational background, 37.0% held a master's degree, 33.8% had a professional OR nursing degree and 26.4% held a bachelor's degree. Only 0.7% had a doctoral degree, and 2.1% reported other forms of education.

In terms of professional experience, 63.4% of participants had more than 11 years of experience in healthcare. Specifically, 25.7% had 11–20 years of experience and 37.7% had over 20 years. (Table 1).

**TABLE 1:** Demographics of the participants.

Total number of operating room nurses *n* = 284	*n*	%
**Education**		
Professional degree^a^	96	33.8
Bachelor's degree	75	26.4
Master's degree	105	37.0
Doctoral degree	2	0.7
Other	6	2.1
**Work experience in healthcare**		
≤ 5 years	33	11.6
5–10 years	71	25.0
11–20 years	73	25.7
≥ 20 years	107	37.7

^a^ Operating room nursing degree.

Self‐assessed expertise in PU management based on Benner's proficiency levels (Benner 1982) revealed that most OR nurses identified as *proficient* (*n* = 142, 50%) or *competent* (*n* = 108, 38%). A smaller proportion identified as *expert* (*n* = 32, 11.3%), and only two nurses (0.7%) considered themselves *novice*. Notably, 87% of participants expressed that further education in PU prevention would be beneficial.

### 5.2 Pressure Ulcer Knowledge Assessment Tool 2.0–Operating Room (PUKAT 2.0 OR)

Table 2 presents the results from the PUKAT 2.0 OR instrument, summarizing the proportion of correct and incorrect responses across the 14 items. In the *aetiology* domain, over 95% of OR nurses correctly identified the causes of PUs and the biomechanical consequences of a patient sliding in a semi‐upright position. Additionally, 90.1% recognized that body weight is a contributing risk factor.

**TABLE 2:** Operating room nurses' knowledge regarding pressure ulcer prevention.

Total number of operating room nurses, *n =* 284				
	Q		Correct answer *n* (%)	Incorrect answer^a^ *n* (%)
Aetiology	1	What is the cause of pressure ulcers?	272 (95.8)	12 (4.2)
	2	A patient undergoes surgery in a semi‐upright position (e.g., the head of the operating table is at a 60° angle). What happens to the sacrum and ischial tuberosities if the patient slides down?	266 (93.7)	18 (6.7)
	3	Moist skin (due to e.g., incontinence, wound exudate, irrigation fluid or skin disinfection) and increased body temperatures are associated with pressure ulcer development. This statement is…	209 (73.6)	75 (26.2)
	4	Where in the tissue do deep tissue injuries develop during surgery?	108 (38)	176 (62)
	5	What type of patients (in terms of body weight) have an increased risk of developing pressure ulcers?	256 (90.1)	28 (9.9)
Classification and observation	6	You observe a blister on both heels of a patient following a 3‐h surgical procedure. Which statement is correct?	117 (41.2)	167 (58.8)
	7	Which of these pictures is a pressure ulcer Category I?	179 (63)	103 (37)
Prevention	8	CASE: A patient is positioned pre‐operatively in a semi‐Fowler position (the individual is supine and the head of the operating table is elevated). Which statement is correct?	146 (51.4)	138 (48.6)
	9	A patient undergoes surgery in a side‐lying position (e.g., hip surgery, lung surgery). Which positioning angle during surgery is associated with the highest risk of developing a pressure ulcer at the trochanter major?	148 (52.1)	136 (47.9)
	10	How should surgical linen be used to prevent pressure ulcers?	161 (56.7)	123 (43.3)
	11	CASE: Your patient is lying on a pressure‐redistributing OR mattress. Do you take other measures to prevent pressure ulcers on the heels?	116 (40.8)	156 (59.2)
	12	Why is repositioning necessary to prevent pressure ulcers?	186 (65.5)	98 (34.5)
	13	Which statement is correct?	193 (68)	91 (32)
	14	Indicate the location on the body where babies have the highest risk of developing a pressure ulcer.	203 (71.5)	81 (28.5)

Abbreviations: OR, operating room; Q, question.

^a^ Incorrect answers and the ‘I don't know’ item were merged to create a single category for ‘incorrect answer’.

However, knowledge gaps were evident in several areas. For example, only 38% correctly identified where deep tissue injuries develop during surgery, and only 41.2% correctly classified heel blisters postoperatively. In the *prevention* domain, responses to questions regarding the use of pressure redistributing devices and positioning strategies showed lower accuracy, with nearly 60% answering incorrectly regarding heel protection measures.

### 5.3 Attitudes Toward Pressure Ulcer Prevention—Operating Room (APuP‐OR)

Overall, the OR nurses demonstrated positive attitudes towards PU prevention. A large majority (94.7%) reported feeling confident in their ability to prevent PUs, and 98.6% agreed that PU prevention should be a priority and a personally important responsibility. Furthermore, 94% acknowledged the financial burden of PUs on society.

The participants strongly disagreed with negatively worded statements. For instance, 93.7% disagreed that the impact of PUs on patients is exaggerated, and 96.5% rejected the notion that too much attention is given to prevention. Additionally, 95.8% disagreed that PUs rarely cause discomfort, and 96.1% rejected the idea that they are not responsible if a PU develops in their patient (Table 3).

**TABLE 3:** Operating room nurses' attitudes regarding pressure ulcer prevention.

Total (*n* = 284)	Agree *n* (%)	Disagree *n* (%)
Personal competency to prevent pressure ulcers		
(+)^a^ I am well trained to prevent pressure ulcers.	232 (81.7)	52 (18.3)
(+)^a^ I feel confident in my ability to prevent pressure ulcers.	269 (94.7)	15 (5.3)
(−)^b^ Pressure ulcer prevention is too difficult. Others are better than I am.	36 (12.7)	248 (87.3)
Priority of pressure ulcer prevention		
(+)^a^ Pressure ulcer prevention should be a priority.	280 (98.6)	4 (1.4)
(+)^a^ I personally have an important task in pressure ulcer prevention.	280 (98.6)	4 (1.4)
(+)^a^ The financial impact of pressure ulcers on society is high.	267 (94)	17 (6)
Impact of pressure ulcers		
(−)^b^ The impact of pressure ulcers on a patient should not be exaggerated.	18 (6.3)	266 (93.7)
(−)^b^ Too much attention goes to the prevention of pressure ulcers.	10 (3.5)	274 (96.5)
(−)^b^ A pressure ulcer almost never causes discomfort for a patient.	11 (3.9)	272 (95.8)
Confidence in the effectiveness of prevention		
(−)^b^ Pressure ulcers are almost never preventable.	5 (1.8)	279 (98.2)
(+)^a^ Pressure ulcers are preventable in high risk patients.	281 (98.9)	3 (1.1)
Responsibility in pressure ulcer prevention		
(−)^b^ I am not responsible if a pressure ulcer develops in my patient.	11 (3.9)	273 (96.1)
(−)^b^ Pressure ulcer prevention is not that important.	5 (1.8)	279 (98.2)

^a^ Positively worded item.

^b^ Negatively worded item.

Pairwise comparisons revealed a statistically significant difference across years of OR experience in terms of perceived training to prevent PUs. Specifically, OR nurses with > 20 years of experience reported feeling significantly better trained than those with 5–10 years of experience (*p* = 0.049). A similar pattern was observed for perceived confidence in the ability to prevent PUs, with increasing confidence associated with greater work experience. Comparisons showed significant differences between OR nurses with > 20 years of experience and those with 5–10 years of experience (*p* = 0.005), as well as between OR nurses with 11–20 years of experience and those with < 5 years and 5–10 years of experience (*p* = 0.02).

## 6 Discussion

This study investigated OR nurses' self‐assessed knowledge and attitudes regarding intraoperative PU prevention. The findings revealed that while participants demonstrated knowledge in PU aetiology, significant gaps were evident in prevention‐related areas, particularly patient positioning.

Attitudes towards PU prevention were assessed in this study using the APuP‐OR instrument. The results showed that OR nurses reported confidence and responsibility in preventing PUs in OR settings, with attitude scores higher than those reported in a previous study by Khong et al. (2020). Despite positive APuP‐OR attitudes, the overall knowledge level (PUKAT OR 2.0) was insufficient when applying a 90% cut‐off for correct responses; only 3 out of 14 items met this threshold. Even when lowering the cut‐off to 70%, the number of correct responses remained limited. Although lowering the threshold could result in scores that seem more acceptable, it also prompts reflection on whether adjusting the optimal level of knowledge in PU prevention is appropriate. With this in mind, the decision was made to retain the 90% threshold.

In contrast, attitudes towards PU prevention were overwhelmingly positive, with nurses expressing confidence, a sense of responsibility, and recognition of the impact of PUs on patients and healthcare systems. Despite favourable attitudes, the overall level of knowledge indicates potential gaps in certain areas. A targeted education and competency development in PU prevention could be a viable strategy, as also discussed in a recent study (Şengezer and Erdoğan 2026). The findings in the present study underscore the importance of OR nurses possessing a thorough understanding of PU aetiology and intraoperative risk factors, as well as implementing protective measures during surgical procedures.

Our participants demonstrated high levels of knowledge in the domain of PU aetiology, with correct responses ranging from 90.1% to 95.8%. This aligns with previous research emphasizing the role of knowledge in delivering evidence‐based care (Beeckman et al. 2011; Bjurbo et al. 2024; Gunningberg et al. 2015). The use of a validated instrument (PUKAT 2.0 OR) enabled the identification of specific knowledge gaps, particularly in the domain of prevention. Notably, questions related to patient positioning yielded lower correct response rates (51.4%–52.1%); this is consistent with the findings of Sørensen et al. (2016), who reported limited positioning competencies among surgical teams.

A substantial proportion of OR nurses (87%) expressed a desire for further education in PU prevention. This is supported by Brooker et al. (2020), who highlighted the need for ongoing education and competency verification in safe patient positioning. Moreover, recent work by Wevling and Linqvist Leonardsen (2025) emphasizes that surgical positioning is a shared responsibility among the entire surgical team, including OR nurses. The PUKAT 2.0 OR instrument may serve as a valuable tool for identifying knowledge gaps and facilitating team‐based discussions on roles and responsibilities. However, further refinement of this instrument is needed to ensure that it reflects the complexity of pre‐positioning in various surgical procedures while ensuring adequate operative exposure for the surgeon. Additionally, further studies are needed, potentially including in‐depth interviews, to explore factors influencing safe patient positioning, for example, using a structured positioning protocol.

### 6.1 Strengths and Limitations

Survey methodology offers a cost‐effective and efficient means of collecting data from large populations, making it particularly suitable for exploring trends, risk factors and professional competencies in healthcare settings (Sharma, 2021). The use of validated instruments in this study enhances the reliability and consistency of the findings.

Some general limitations must be acknowledged. Self‐reported data are inherently susceptible to recall bias and social desirability bias, which may affect the accuracy of responses (Colbert et al. 2013). Ambiguities in question wording can also lead to misinterpretation and response errors; for example, our study had questions concerning negotiations, which may have confused the participants. The 242 participating OR nurses, representing different regions of Sweden and various hospital types, can be viewed both as a limitation and a strength. These respondents could perhaps be more interested in PU prevention than non‐respondents, and if so, this would have influenced our findings on knowledge and attitudes. Moreover, the low response rate (15%) restricts the generalizability of the findings to the broader international populations.

### 6.2 Implications for Research and Practice

Given that most PUs are preventable, it is imperative to raise awareness and promote evidence‐based prevention strategies within the OR team. Each member of the surgical team holds a responsibility to enhance patient safety during surgery. Further studies are needed to investigate why positive attitudes do not necessarily translate into adequate knowledge or consistent preventive actions. Mixed‐methods research could help clarify underlying factors such as organizational culture, workflow constraints or insufficient training. The observed gap between positive attitudes and insufficient knowledge suggests the need for systematic, evidence‐based educational interventions. Structured training programs, simulation‐based learning and regular competency assessments should be integrated into continuing professional development for OR nurses. These interventions should include all team members, such as surgeons, anaesthesiologists and nurse anaesthetists, to assess their knowledge and attitudes towards PU prevention.

## 7 Conclusion

While OR nurses exhibit positive attitudes towards PU prevention, their knowledge levels remain insufficient. Strengthening evidence‐based education and implementing structured assessment tools is essential for improving intraoperative PU prevention. As PU prevention is a critical patient safety issue, targeted interventions and team‐based strategies should be prioritized in perioperative care.

## Author Contributions

**Karin Falk‐Brynhildsen:** conceptualization, data curation, formal analysis, investigation, methodology, validation, visualization, writing – original draft, writing – review and editing, project administration. **Carina Bååth:** conceptualization, data curation, formal analysis, investigation, methodology, validation, visualization, writing – original draft, writing – review and editing. **Dimitri Beeckman:** conceptualization, data curation, methodology, writing – review and editing. **Christine Leo Swenne:** conceptualization, writing – review and editing. **Charlotte Raepsaet:** conceptualization, writing – review and editing. **Camilla Wistrand:** conceptualization, data curation, formal analysis, investigation, methodology, validation, visualization, writing – original draft, writing – review and editing.

## Funding

The authors have nothing to report.

## Ethics Statement

Ethical approval was obtained from the Swedish Ethical Review Authority (registration number: 2020–01212). Participants received written information outlining the study's purpose and procedures, and the voluntary nature of participation. The survey link was distributed via email, and completion of the questionnaire was considered as informed consent. Participant confidentiality was ensured, with data stored securely on Ghent University servers protected by firewalls in compliance with the Swedish General Data Protection Regulation (GDPR).

## Conflicts of Interest

The authors declare no conflicts of interest.

## Data Availability

The data that support the findings of this study are available from the corresponding author upon reasonable request.
